# Saliva Cortisol in Girls With Functional Abdominal Pain Disorders: A Randomized Controlled Dance and Yoga Intervention

**DOI:** 10.3389/fped.2022.836406

**Published:** 2022-05-12

**Authors:** Elin Areskoug Sandberg, Anna Duberg, Ulrika Lorenzon Fagerberg, Evalotte Mörelius, Stefan Särnblad

**Affiliations:** ^1^University Health Care Research Center, Faculty of Medicine and Health, Örebro University, Örebro, Sweden; ^2^Center for Primary Health Care Research, Department of Clinical Sciences, Lund University, Malmö, Sweden; ^3^Centre for Clinical Research, Department of Paediatrics, Västmanland Hospital, Region Västmanland, Uppsala University, Västerås, Sweden; ^4^Department of Women’s and Children’s Health, Karolinska Institutet, Stockholm, Sweden; ^5^School of Nursing and Midwifery, Edith Cowan University, Joondalup, WA, Australia; ^6^Perth Children’s Hospital, Nedlands, WA, Australia; ^7^Department of Pediatrics, Faculty of Medicine and Health, Örebro University, Örebro, Sweden

**Keywords:** cortisol, yoga, dance, children, stress, intervention, just in time, recurrent abdominal pain

## Abstract

**Introduction:**

Functional abdominal pain disorders (FAPDs) are common among girls and has been associated with stress. Cortisol is one of the major stress hormones. Dance and yoga have been shown to reduce abdominal pain among girls with FAPDs.

**Aim:**

To investigate the effect of an 8-month intervention with dance and yoga on cortisol levels in saliva among girls with FAPDs.

**Methods:**

A total of 121 girls aged 9–13 years with irritable bowel syndrome (IBS) or functional abdominal pain were included in the study. Participants were randomized into an intervention group and a control group. The intervention group attended a combined dance and yoga session twice a week for 8 months. Saliva samples were collected during 1 day, in the morning and evening, at baseline, and at 4 and 8 months. Subjective pain and stress were assessed as well.

**Results:**

No significant effects on saliva cortisol levels between groups were observed after completion of the intervention at 8 months. However, evening cortisol and evening/morning quotient were significantly reduced at 4 months in the intervention group compared to the control group (*p* = 0.01, *p* = 0.004). There was no association between cortisol quota and pain or stress.

**Conclusion:**

Improvements in cortisol levels were seen in the intervention group at 4 months but did not persist until the end of the study. This indicates that dance and yoga could have a stress-reducing effect during the ongoing intervention.

## Key Notes

• Functional abdominal pain disorders are common in girls and often associated with emotional distress. Cortisol in saliva is an indicator of stress.
• This randomized controlled dance and yoga intervention showed no significant effects on cortisol levels between the study groups at the end of the 8-month intervention.
• Significant differences in saliva cortisol levels at 4 months between the intervention and the control group indicate a positive stress-reducing effect during the intervention.

## Introduction

Functional abdominal pain disorders (FAPDs) are common conditions among children worldwide and are often associated with stress (1). As many as 16.4% of school children report FAPDs (1), when diagnosed according to the ROME III criteria and defined as chronic or recurrent abdominal pain not explained by underlying organic disorders (2). For unknown reasons, the prevalence among girls is significantly higher than that among boys (1, 3).

The association between psychological aspects and recurrent abdominal pain has been known for a long time (4). Emotional distress is often reported among patients with FAPDs (3), and stress and traumatic life experiences increase the prevalence of these disorders (1). Not only do FAPDs interfere with functions in daily life, recurrent pain among youths is also associated with internalizing symptoms (5) as well as depression and anxiety (3) and leads to an increased risk of mental health problems in future life (3, 6).

Cortisol is one of the major stress hormones, and because of its manageability through sampling in saliva, it has been extensively used in studies (7). The release of cortisol follows a diurnal pattern, with a high release during the morning and a reduction during the day (8). During long-term stress, the diurnal curve is flattened due to higher evening values (9). Changes in cortisol secretion over time are associated with disease and negative health outcomes (10). Due to its importance regarding the growth, maturation and development of the brain, healthy cortisol levels are especially important during childhood (10). As with many other stress-related diseases, alterations in cortisol levels have previously been observed among children with FAPDs, such that these children present higher total cortisol secretion than healthy children (11).

Despite the high prevalence of FAPDs, effective treatments are still rare (12). New pharmaceuticals are being tested, however, up to date there is no conclusive evidence regarding pharmaceuticals (12, 13). A multimodal and complex etiology of FAPDs has been recognized, and the use of a biopsychological model when designing interventions has grown in popularity over the last years (12). Psychological treatments, including cognitive behavioral therapies can help children cope with and reduce pain (13). In addition, many new psychological interventions like acceptance and commitment therapy as well as mindfulness are under development and research (13). Physical activity has shown beneficial effects in the practical management of FAPDs (14), by improving function and distraction from pain. Dance is one of the most popular physical activities among girls (15) and has been shown to improve both physiological (16) and psychological (17, 18) health in youths. Yoga, with its focus on physical posture, controlled breathing and attention, has been shown to be effective in reducing anxiety and stress and increasing musculoskeletal function among children and adolescents (19, 20). In addition, yoga has been showing promising results regarding reducing anxiety and depression (21), alleviating abdominal symptoms among children and adolescents with IBS (22), and having a direct effect on salivary cortisol levels in both sexes at different ages (23, 24). Together, dance and yoga complement each other in that dance involves dynamic, rhythmic physical activity, while yoga enhances relaxation, and focus (25). In this article, we study the effects of an 8-month long randomized controlled trial with a combined dance and yoga intervention for girls with FAPDs, the Just In TIME study. Previous results of the same intervention, showed reduction of abdominal pain during and after the intervention (26), and thereby holds promise for being a future alternative treatment for FAPDs among girls.

Alterations in cortisol levels in children with FAPDs have been previously described; however, interventions observing changes in cortisol levels over time are limited. Thus, this study aims to investigate the effects on diurnal cortisol secretion during and after an 8-month intervention of dance and yoga among girls 9–13 years old with FAPDs (i.e., functional abdominal pain and irritable bowel syndrome). Our hypothesis is that the intervention would result in an increase in morning cortisol value, a decrease in evening values and a reduced evening to morning quotient at 8 months. Since knowledge regarding cortisol levels and FAPDs in general is limited, baseline values for the whole studied group contribute to the sparse amount of research in this field.

## Materials and Methods

**FIGURE 1:**
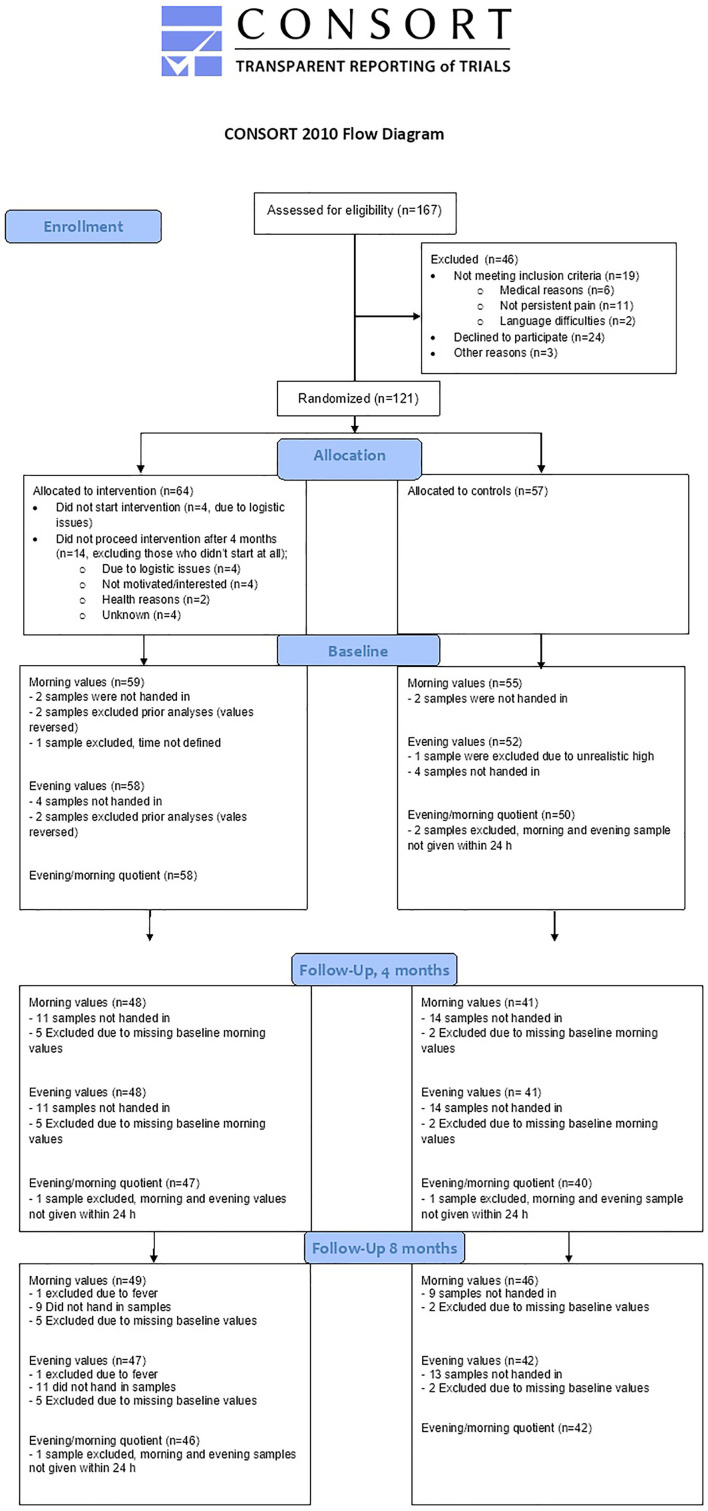
Consort flow chart.

The study was performed as a prospective randomized controlled study with one intervention group and one control group (Figure 1). This study was a part of the larger study “Just in TIME” (Try, Identify, Move, and Enjoy) that investigated several effects of dance and yoga on FAPDs. Clinical trial registration number: NCT02920268. This trial has been previously described in a study protocol (27) and also regarding effects on abdominal pain (26).

### Recruitment of Participants

The participants were girls 9–13 years old diagnosed with FAPDs or IBS with persistent pain after examination at a pediatric center. As the Rome III criteria were current at the start of the inclusion, these criteria were used for irritable bowel syndrome (IBS) and functional abdominal pain (FAPDs) for consistency throughout the study (2). Recruitment of participants was made from the local registry of diagnoses at the Department of Pediatrics in Örebro and Västerås, from primary and school health care in the Region of Örebro and Västerås and through advertisements in Västmanland and Örebro County. The recruitment process is further described in earlier publications (26, 27). After written consent was received from the legal guardians, the diagnosis according to the inclusion criteria was verified in the medical records and/or by examination by a pediatrician. Subjects were excluded if they had celiac disease and/or inflammatory bowel disease (IBD) as a comorbidity. Further exclusion criteria were difficulties following instructions, severe psychiatric symptoms demanding other treatments, and current treatment with cognitive behavioral therapy (CBT).

Girls and their legal guardians who consented to participate in the study and were eligible for the study according to the inclusion criteria completed the baseline measurement. Baseline data was collected via questionaries administered in an auditorium at both sites after school hours. Members from the project team were present to measure height and weight, provide assistance and answer questions if needed. The full questionnaire is further explained in the study protocol (27). The participants filled in pain diaries at home during a 1-week period at baseline. To be included, abdominal pain had to be with a severity of at least 4 or higher according to the Faces Pain Scale-Revised (FPS-R) (total range 1–20) (28). Girls scoring 13 or more on the Children’s Depression Screener (ChilD-S) (29) were offered an appointment with a psychologist to assess whether further support was needed and whether participation in the study was appropriate. An external statistician performed the randomization based on site, pain intensity and age at baseline. The randomization process is described in detail in the study protocol (27). Information about group affiliation was sent out by mail.

### Intervention

The dance and yoga classes were performed as a group activity twice a week for 8 months. The intervention was performed after school hours for 60 min per session (dance 30 min, yoga and relaxation 25 min and reflection 5 min), as described in the Study Protocol (27). Each group included 7–14 girls and was led by experienced and purposely educated instructors, one or two at each time. All instructors had a former education in either health care or pedagogy. The intervention was guided by three instructors at each site. The instructors alternated during the intervention time period but one or two instructors were always present at each class. The dance section included a warm-up session with up-tempo music and captivating rhythm to engage large muscle groups and a short, choreographed section led by the instructor. The focus was on socialization and enjoyment rather than performance. The yoga consisted of playful movements such as creative storytelling with a focus on breathing and attention (performed both individually, in pairs and as a group). Yoga postures focusing on massage of the abdomen as well as balancing postures were carefully selected. Both centers followed the same routine. The control group was requested to live as usual, and health care was available for everyone when needed.

### Data Collection

Saliva cortisol samples were collected at home with a Salimetrics polymer swab two times during a school day [at 07.00 am and 08.00 pm (±1 h)] at baseline and at 4 and 8 months. The girls were instructed to provide the morning sample shortly after awakening while still in bed. Evening samples were provided at least 1 h after food intake, intake of liquids, and tooth brushing to avoid contamination. Samples were either picked-up in the girls’ homes by members in the research group or sent to the lab in prepaid padded envelopes. All saliva samples were centrifuged upon arrival to the laboratory in Örebro and subsequently stored at −20°C pending analysis. When all samples were collected, they were sent on dry ice in an unbroke freezing chain to the laboratory at Linköping University Hospital for analysis (9). Collection of saliva samples was given during the same period of time for both the intervention and control groups.

Abdominal pain was measured with FPS-R (28, 30) three times a day during 1 week at baseline as well as at 4 and 8 months. Clinical responses were counted if the mean value in the diary showed a mean decrease of two steps or more during the week of measure (31). To assess the subjective stress level, the participants answered the question; “how often have you experienced stress over the last week?” on a 5-level scale (0 = always, 1 = often, 2 = sometimes, 3 = rarely, and 4 = never) in the questionnaire administered at baseline and at each follow up.

### Biochemical Analysis

The samples were analyzed using a commercial enzyme immunoassay method (Salivary Cortisol Enzyme Immunoassay Kit; Salimetrics LLC). A Tecan robot method was used, modified to suit a 25-μL saliva volume. The intra assay coefficient of variation (CV) was 5%, and the total CV was 7.7 and 7.5% for low and high values, respectively (32).

### Statistics

All data were analyzed using SPSS (Statistical Package for the Social Sciences, version 24, IBM Corp., Armonk, NY, United States). A *p*-value of <0.05 was considered statistically significant. Cortisol levels are presented as the median with interquartile range (IQR) in Table 2. Calculations were performed as intention to treat as well as per protocol where a cut of was set as 50% attendance to the study. Since the outcome of the per protocol only resulted in minor differences compared to the intention-to-treat values, only intention-to-treat analyses are presented in this article. All parameters were tested for normal distribution. In Table 1, an independent *t*-test was used on continuous data to evaluate any associations or differences between groups, and the chi-square test was used on categorical data. Cortisol data were analyzed with non-parametric tests since the distribution was skewed. The Mann–Whitney *U* test was used for comparisons between groups, and the Wilcoxon signed-rank test was used for comparisons between different time points within groups, as shown in Table 2. Evening/morning quotient was calculated by dividing each participant’s evening value by the morning value. This calculation gives a quota between 0 and 1, which adjusts for variances in individual baseline values, a method that has been used before (33). A smaller quota indicates a more pronounced diurnal rhythm. To compare whether participation rate influenced the evening/morning quotient, the intervention group was divided into three sub-groups depending on the girls’ presence (low attendance 0–49%, medium attendance 50–74%, high attendance 75–100%) and potential differences were calculated using the Kruskal–Wallis test. Saliva cortisol were also analyzed in the total population in regards of clinical outcome, meaning a decrease of 2 or more in the FSP-R assessment. Median as well as IQR of the evening/morning cortisol quota was calculated in the group with decreased abdominal pain versus no decrease in abdominal pain. Stress levels were compared between groups as percentages as well as median (IQR). Differences between the groups in regards of stress and pain were measured with Mann–Whitney *U* test. A power calculation was performed for the primary outcome abdominal pain (26, 27). The methodology followed the consort checklist.

**TABLE 1:** Baseline characteristics.

	Intervention *n* = 59	Control *n* = 55
**Age at start (year)**		
Mean (SD)	10.4 (1.37)	10.7 (1.32)
**Diagnose, *n* (%)**		
IBS	25 (42%)	19 (35%)
FAP	34 (58%)	36 (65%)
**Height, (*m*)**		
Mean (SD)	1.47 (0.10)	1.47 (0.09)
**Weight class, *n* %**		
Underweight	5/56 (9%)	4/51 (8%)
Normal	36/56 (64%)	40/51 (78%)
Overweight	8/56 (14%)	5/51 (10%)
Obese	7/56 (12.5%)	2/51 (4%)
**BMI (kg/m^2^)**		
Mean (SD)	19.18 (4.03)	18.17 (3.02)
**Menarche *n* (%)**		
Yes	5/58 (9%)	6/55 (11%)
No	53/58 (91%)	49/55 (89%)
**ChilD-S**		
Mean (SD)	8.68 (4.265)	7.98 (4.357)
Median (IQR)	9.0 (5.50–11.00)	8.0 (5.00–10.00)
**Self-rated health**		
Mean (SD)	2.8 (0.55)	2.75 (0.726)
Median (IQR)	3.0 (2.00–3.00)	3.0 (2.00–3.00)
**Reported stress**		
Never	7/59 (12%)	9/55 (16.5%)
Rarely	16/59 (27%)	14/55 (25.5%)
Sometimes	20/59 (34%)	21/55 (38%)
Often	11/59 (18.5%)	11/55 (20%)
Always	5/59 (8.5%)	0/55 (0%)
Median (IQR)	2 (1–3)	2 (2–3)

**TABLE 2:** Saliva cortisol levels at baseline and during the intervention.

	Intervention	Control	*p*-value
**Morning values**			
*n*	59	55	
Baseline median, (IQR)	10.9 (8.8–14.2)	10.1 (7.6–13.3)	0.108
*n*	48	41	
4 months median, (IQR)	11.2 (7.2–16.3)	9.5 (7.3–12.4)	0.055
*P*-value (compared to baseline)	0.72	0.91	
*n*	49	46	
8 months median, (IQR)	5.7 (2.9–10.2)	6.9 (4.6–11.2)	0.375
*P*-values (compared to baseline)	<0.001	0.019	
**Evening values**			
*n*	58	52	
Baseline median, (IQR)	1.1 (0.9–1.8)	1.1 (0.8–1.5)	0.710
*n*	48	41	
4 months median, (IQR)	0.9 (0.6–1.2)	1.2 (0.8–2.2)	0.010
*P*-value (compared to baseline)	0.055	0.428	
*n*	47	42	
8 months median, (IQR)	0.9 (0.6–1.5)	1.0 (0.7–1.4)	0.776
*P*-value (compared to baseline)	0.555	0.179	
**Evening/morning quotient**			
*n*	58	50	
Baseline median, (IQR)	0.111 (0.08–0.15)	0.107 (0.07–0.18)	0.753
*n*	47	40	
4 months median, (IQR)	0.087 (0.05–0.15)	0.142 (0.09–0.26)	0.004
*P*-value (compared to baseline)	0.249	0.140	
*n*	46	42	
8 months median, (IQR)	0.167 (0.08–0.43)	0.143 (0.09–0.22)	0.491
*P*-value (compared to baseline)	<0.001	0.199	

*All saliva cortisol levels are presented in nmol/L. The median and IQR were used since the data distribution was skewed. Saliva cortisol levels between the intervention and control groups were compared with the Mann–Whitney U test, and the Wilcoxon signed-rank test was used for comparisons between different time points within groups.*

### Missing Data

Samples were missing due to different reasons. Only participants who handed in morning cortisol samples at baseline were included in the analyses. In cases where samples were not given within 24 h, morning and evening levels were analyzed, but the quotients were not calculated. Drop-outs and missing samples are presented in flow chart Figure 1.

### Ethical Considerations

The study follows the guidelines for biomedical research according to the Helsinki declaration. The Regional Ethics Committee of Uppsala, Sweden, approved the study (dnr 2016/082/2). All information about participants was registered according to the General Data Protection Regulation (GDPR). All children agreed upon participation, and written consent was received from parents/caregivers before inclusion. Information and data obtained from the participants were coded, and the code key was held at the University Health Care Research Center, Region Örebro County, Sweden.

## Results

Study group characteristics at baseline are presented in Table 1. The mean age at the start of the intervention was 10.4 years (SD = 1.4) in the intervention group and 10.7 years (SD = 1.3) years in the control group. There were no differences in morning or evening cortisol levels between groups at baseline, median (IQR) 10.9 (8.8–14.2)/1.1 (0.9–1.8) nmol/L among girls in the intervention group versus 10.1 (7.6–13.3)/1.1 (0.8–1.5) nmol/L among girls in the control group (*p* = 0.108/*p* = 0.710) (Table 2). The median (IQR) morning and evening values for all included participants were 10.5 (7.9–13.9) and 1.1 (0.8–1.5) nmol/L, respectively.

### Changes in Morning and Evening Cortisol Values

The evening value was significantly lower in the intervention group at the 4-month follow-up than in the control group, with a median (IQR) of 0.9 (0.6–1.2) nmol/L compared to 1.2 (0.8–2.2) nmol/L (*p* = 0.01), indicating lower stress in the intervention group during the intervention. However, the difference to baseline values was not statistically significant within the intervention group (*p* = 0.055). Furthermore, there was no significant difference between the groups regarding morning cortisol levels at 4 months. At the 8-month follow up, there were no differences between groups regarding the morning or evening values. The within-group morning cortisol levels were significantly reduced at 8 months compared to baseline in both groups; values in the intervention group median (IQR) 5.7 (2.9–10.2) nmol/L (*p* ≤ 0.001) and in the control group median (IQR) 6.9 (4.6–11.2) nmol/L (*p* = 0.019).

### Changes in Evening/Morning Quotient

**FIGURE 2:**
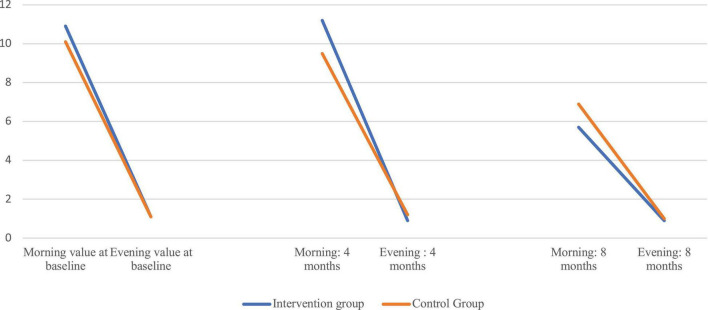
Graphic illustration of morning and evening cortisol levels (nmol/L) at baseline, 4 and 8 months in the intervention and control groups.

The evening/morning cortisol quotient at 4 months was significantly lower in the intervention group, median (IQR) 0.087 (0.05–0.15) nmol/L, than in the control group, median (IQR) 0.142 (0.09–0.26) nmol/L (*p* = 0.004). The difference did not remain until the end of the study. At 8 months, the evening/morning quotient increased in both groups. The evening/morning quotient was significantly higher at 8 months than at baseline in the intervention group (*p* < 0.001). The changes in evening/morning quotient over time is illustrated in Figure 2.

### Adherence to the Study

At the first half of the intervention (autumn), 8 girls had low attendance, 14 girls had a medium attendance, and 25 girls had a high attendance (*n* = 47). At the second half of the intervention (spring), 16 girls had low attendance, 13 girls had medium attendance and 18 girls had high attendance (*n* = 47). During the whole intervention period (both the autumn and spring) period, 12 girls had low attendance, 17 girls had medium attendance and 18 girls had high attendance (*n* = 47). There was no statistical difference in the median evening/morning quotient between low, medium, and high attendance group at 4 months (*p* = 0.919) or at 8 months (*p* = 0.368).

### Saliva Cortisol in Relation to Abdominal Pain

No significant difference in evening/morning saliva cortisol quota were observed between the group with decreased abdominal pain compared with no decrease of abdominal pain. The median (IQR) cortisol quotient was 0.14 (0.075–0.264) in the group with decreased abdominal pain vs. 0.11 (0.064–0.196) (*p* = 0.452) in the group with no decrease of abdominal pain at 4 months and 0.18 (0.082–0.538) vs. 0.15 (0.087–0.267) (*p* = 0.460) at 8 months.

### Reported Stress Levels

There were no differences in reported stress between the groups at baseline, 4 months or 8 months. Median (IQR) stress levels were 2 (2–3) in the intervention group and 3 (2–3) in the control group at 4 months and 3 (2–3) in the intervention group and 3 (2–3.5) in the control group at 8 months.

## Discussion

The aim of this study was to investigate the effect of a dance and yoga intervention for girls with FAPDs (IBS and FAP) regarding cortisol in saliva. Even though this intervention showed positive effects with regards to abdominal pain (26), no conclusive differences in cortisol were observed in this study after 8 months. However, improved cortisol levels were observed in the intervention group at 4 months, with significantly lower evening values and reduced evening/morning quotients in the intervention group than in the control group. Overall, this finding points toward a positive effect of the intervention at 4 months.

First, it is worth acknowledging that the general knowledge regarding saliva cortisol levels in this target group is limited. Two previous studies have pointed toward increased morning cortisol and higher total cortisol secretion among children with FAPDs (11, 34). Although stress is considered a contributing factor for FAPDs, few studies have investigated cortisol levels in this target group. Even though the changes in cortisol were not statistically significant at the end of intervention, our measured baseline levels contribute to a broader understanding of FAPDs in this field of research. The median baseline level in our study population was 10.5 nmol/L in the morning and 1.1 nmol/L in the evening, which all represent normal reference levels according to previously described measures from healthy subjects in the same age group: 4–27 nmol/L at 08.00 h and 0.7–7 nmol/L at 23:00 h (35), and follows a healthy diurnal pattern (8). Normal cortisol levels already at start make the potential room for improvement small, and the possibility of achieving significant changes is reduced. One important factor was that all baseline samples were collected after the girls’ summer vacation, which potentially reduced stress and might have influenced the baseline cortisol levels toward normalization. A third control group with healthy children would be needed to evaluate this further.

Significant positive changes in saliva cortisol levels were observed in the intervention group at 4 months compared to the control group. At the 4-month follow-up, subjects in the intervention group showed significantly lower evening values resulting in a reduced evening/morning quotient with a larger difference between morning and evening levels. Overall, improvement at 4 months pointed toward healthier cortisol secretion in the intervention group. This indicates a positive effect of dance and yoga during the time of the intervention. However, at 8 months, the results were reversed and even pointed toward an increased cortisol quotient among girls in the intervention group.

As presented previously, abdominal pain decreased significantly in the intervention group throughout the study (26). Yet, no difference in median evening/morning cortisol quota was found between the participants with decreased abdominal pain compared with study subjects without decrease in abdominal pain. Neither did we found a significant difference between groups in regards of perceived stress. An association between perceived stress and recurrent pain has been described among girls before (36). Less is clear about the relationship between pain and saliva cortisol. Correlations between acute pain and saliva cortisol reactivity are common during infancy but tend to diminish with increasing age (37). Similarly, recurrent pain seems to lack a correlation to saliva cortisol in adolescents (38). This is in line with our results and the reason could be that saliva cortisol and subjective stress and pain assessments measures different perspectives of an individual’s experience and perception. Another explanation could be that pain was measured over a week and not in immediate conjunction with saliva sampling.

On the other hand, it has been shown that high cortisol levels might be associated with introvert behavior, whereas low cortisol levels are associated with extrovert behavior (34). FAPDs are commonly associated with internalizing behavior (5), which thereby could explain one potential reason of the elevated cortisol levels in the target group previously described (11, 39). One possible explanation of improved cortisol levels might be due to emotional expression and activation and thereby decreased burden of internalizing behaviors. At 8 months, the morning values were significantly lower in both groups, which might indicate an elevated level of stress the day before the saliva sampling, which potentially can negatively affect the result (40, 41). The 8-month follow-up took place during May-June, a stressful time for all students. This fact, however, did not differ between groups. Toward the end of the intervention, it is possible that the girls in the intervention group also experienced elevated stress and increased worriedness due to the uncertainty of the time after the intervention. Breaking up a safe and social group might increase feelings of loneliness, which can lead to stress and affect cortisol levels in a negative manner (42). The 8-month samples were collected during the same week as the intervention ended. To address this, it would be of interest to measure cortisol values just before the end of the study, as well as some weeks after completion to address the long-term effects not affected by the immediate break-up of the group.

There could be a number of reasons explaining why the attendance rate dropped over time. The intervention was 8 months long, which could constitute a logistic challenge over time for the parents and families, and moreover, the intervention was given twice weekly, and some of the girls may have experienced that participation once a week gave sufficient effect for them during the second half of the intervention. However, no significant differences in the evening/morning quotient at 4 or 8 months were observed between the sub-groups with low, medium, or high attendance.

Even though cortisol sampling in saliva is frequently used in research covering stress and HPA-axis function among children, there is a lack of gold standard regarding methodology and presentation of values, which contributes to challenges when interpreting and comparing studies to each other (43). Diurnal salivary secretion can be measured and presented in different ways. The method we have used is based on the evening/morning quotient, to adjust for baseline variance among individuals. Because of the diurnal changes and daily fluctuations, it can be misleading to compare individual values against each other without taking into account its broader context. Intraindividual changes might be of greater interest to study than reference values.

Age affects cortisol levels, especially around puberty (11). Our measurement took place during a critical period in an age when hormonal changes were present. To rule out the effect on cortisol due to the stage of puberty, this needs to be adjusted for with specificity. Such adjustments were not performed in our measurements. However, age and menarche were comparable in our study groups, and we assume that the hormonal effect due to puberty was similar in both groups. A larger number of girls in the intervention group had obesity and overweight compared to the girls in the control group but there was no significant difference in mean BMI. Because of the small number in each of the four different weight classes no effort was made to make comparisons in evening/morning quotient between groups. There is no consistent evidence regarding the relationship between obesity and cortisol levels in children. One previous study has indicated that salivary cortisol has a negative association with BMI in children with functional abdominal pain (11). However, that study had a small sample size and needs to be reproduced before any conclusions can be made.

Cortisol levels in childhood is complex, and it should be noted that dance and yoga might be one way of many to potentially influence this in a positive direction. Early childhood experience, peer and family relationships, school environment as well as nutrition, pharmaceuticals and supplements might as well play an important role in stress and cortisol regulation in children. Recently, intake of probiotics has shown a positive effect on cortisol levels among healthy children (44). However, use of pharmaceuticals, diet regulation and supplements were beyond the scoop of this study.

### Strengths and Limitations

This study contributes to filling the research gap regarding cortisol levels among children with FAPDs. Methodological strengths of the study include the randomized controlled design, long intervention time period, and the use of an objective measurement to provide new insights and increase understanding of this vulnerable target group and a potentially health-strengthening intervention. Saliva samples were self-administered and collected at home, which should lower the level of stress related to the sampling procedure. However, we cannot verify how meticulously the participants followed the prescribed guidelines.

Some methodological limitations should be addressed and can be improved in further studies. First, the measures used in this study included only two saliva samples per day, which does not allow us to examine the cortisol awakening response, daily fluctuations or obtain clarity in how cortisol was released during the day. Multiple measures would provide further important information about cortisol secretion. However, multiple measurements are logistically more difficult to carry through, especially among children, and comes with a greater uncertainty about given samples. Second, single-day sampling does not reveal information about cortisol secretion over an extended period of time. Another way to evaluate the effect of the intervention had been to analyze cortisol in hair (45). However, cortisol analyses in hair do not provide any information regarding current cortisol levels, nor does it rule out diurnal variation and the evening/morning quotient. Preferable measurements should be performed in both saliva and hair to evaluate cortisol levels over extended time periods as well as daily fluctuations.

The number of participants is another limitation in this study. Even though the drop-out rate was fairly low, the participation rate dropped over time. Results could possibly have been more pronounced if the participation rate continued high throughout the study. The power calculation was performed on the primary outcome for the whole study, which was abdominal pain. It is possible that the power is underestimated and that some results would differ if the sample size was larger. Future research is needed to confirm results found in this article and to further generalize trial findings into other target groups.

## Conclusion

Findings from this study did not reveal any significant changes in cortisol levels measured after the completion of the intervention. However, significant improvements at 4 months can reflect an effect on cortisol levels during ongoing intervention. Effective treatment strategies for girls with FAPDs are needed, and objective measures such as cortisol can contribute to a wider understanding of the physiological effects of interventions in this target group in short- and long-term follow-ups. This study contributes to the growing understanding and knowledge of active non-pharmacological treatments for this target group.

## Data Availability Statement

The raw data supporting the conclusions of this article will be made available by the authors, without undue reservation.

## Ethics Statement

The studies involving human participants were reviewed and approved by the Regional Ethics Committee of Uppsala, Sweden (dnr 2016/082/2). Written informed consent to participate in this study was provided by the participants’ legal guardian/next of kin.

## Author Contributions

EAS performed the statistical analysis together with SS and wrote the first draft. SS and EM were responsible for the analyze process and data, and were guarantors of the work. SS, UL, and EAS contributed with medical evaluation and data collection. AD coordinated the study and intervention. AD, UL, SS, and EM were steering committee members in the study, contributed to the design of the study and writing of the manuscript, and read and approved the final manuscript. All authors interpreted the data and revised the draft critically for important intellectual content.

## Conflict of Interest

The authors declare that the research was conducted in the absence of any commercial or financial relationships that could be construed as a potential conflict of interest.

## Publisher’s Note

All claims expressed in this article are solely those of the authors and do not necessarily represent those of their affiliated organizations, or those of the publisher, the editors and the reviewers. Any product that may be evaluated in this article, or claim that may be made by its manufacturer, is not guaranteed or endorsed by the publisher.

## Abbreviations

CBT: cognitive behavioral therapy
FAP: functional abdominal pain
FAPDs: functional abdominal pain disorders
FPS-R: Faces Pain Scale-Revised
IBS: irritable bowel syndrome.
